# Effectiveness of IV Ketamine Therapy in the Management of Treatment Resistant Depression

**DOI:** 10.1192/bjo.2026.11855

**Published:** 2026-06-30

**Authors:** Imtiaz Ahmad Dogar, Palvisha Sajid, Dua Fatima, Sammar Fatima, Fatima Tuz Zahra

**Affiliations:** 1 Faisalabad Medical University, Faisalabad, Pakistan; 2 DHQ/Allied Hospitals, Faisalabad, Pakistan

## Abstract

**Aims:**

In the light of limited relevant local literature, we planned our research to build a scientific database related to the immediate and sustained effects of this innovative treatment modality at our tertiary care hospital, in order to start ketamine clinic in our department . We didn’t find any research in Faisalabad, Pakistan on the subject, ensuring a more in depth understanding of the tolerability and acceptability of ketamine therapy in our patient population. The research findings will provide an insight into the effective use of ketamine therapy in cases of TRD

**Methods:**

It was a non randomized trial held in department of psychiatry DHQ/ Allied Hospitals Faisalabad Medical University, Faisalabad, Pakistan.

Sample size : 30 ( purposive sampling)

The study started after approval from ethical review committee of FMU, Faisalabad. The patients fulfilling inclusion criteria were enrolled and informed consent taken. All patients received detailed informational care session about nature of IV ketamine therapy. IV ketamine was given in subanesthetic dose of 0.5mg/kg body weight in 100ml normal saline infusion over 60 minutes after an overnight fast and under supervision of an anesthetist. Pulse, Blood pressure and oxygen saturation was monitored during infusion to ensure safety . Patient was kept in recovery until fully conscious . 8 Treatment sessions were administered over 4 weeks . Patients were assessed using Hamilton Depression Rating Scale (HAM-D) after each dose, then 2 weeks and finally 1 month after last dose.

A 50% reduction in HAM-D scores at 1 hour after 1^st^ dose was considered as treatment response.

**Results:**

Regarding treatment outcomes, IV Ketamine therapy demonstrated promising results. Out of 30 patients, 22 (73.3%) showed a positive response to ketamine therapy, whereas 8 patients (26.7%) didn’t respond. This finding indicates a relatively high efficacy rate of ketamine in managing TRD within the studied group

**Conclusion:**

This study demonstrates that IV ketamine at a sub anesthetic dose produces rapid and substantial improvement in depressive symptoms among patients with TRD. The response rate of 73.3% is consistent with the global data, reinforcing ketamine’s role as a transformative intervention in modern psychiatric care.The persistence of symptom improvement at two weeks and one month follow up assessments suggests that repeated IV ketamine administration may offer short to medium term benefits in TRD Future researches with larger sample size, longer follow up and controlled designs will be essential to validate these findings and optimize clinical protocols for widespread implementation

